# Modified Lemaire extra-articular stabilisation of the knee for the treatment of anterolateral instability combined with diffuse pigmented villonodular synovitis: a case report

**DOI:** 10.1186/s12891-018-2248-7

**Published:** 2018-09-11

**Authors:** Cliodhna Farthing, Gernot Lang, Matthias J. Feucht, Norbert P. Südkamp, Kaywan Izadpanah

**Affiliations:** Department of Orthopedic and Trauma Surgery, Medical Center - Albert-Ludwigs-University of Freiburg, Faculty of Medicine, Albert-Ludwigs-University of Freiburg, Freiburg im Breisgau, Germany

**Keywords:** PVNS, Tenodesis, Anterolateral instability, Lemaire procedure

## Abstract

**Background:**

Diffuse pigmented villonodular synovitis (PVNS) of the knee is a rare proliferative joint disease associated with high recurrence rates following surgical treatment. Intra-articular joint instability in conjunction with PVNS implies complex reconstructive strategies due to the destructive nature of the disease.

**Case presentation:**

Here, we present the case of a young patient with refractory PVNS and a chronic ipsilateral anterior cruciate ligament (ACL) rupture. Clinically, the patient presented with a grade 3 pivot shift phenomenon, indicating anterolateral rotational instability. Usually, PVNS implies a contraindication for ACL reconstruction due to the degenerative and pro-inflammatory joint microenvironment that is induced and maintained by PVNS. Therefore, we have performed a modified Lemaire extra-articular stabilization resulting in significant clinical improvement and subjective joint stability. In the latest follow-up examination at 12 months, the patient reported subjective joint stability and no swelling. In the clinical examination, the patient showed dynamic joint stability during walking. Additionally, the patient presented with grade 0 in pivot-shifting compared to the contralateral knee. The Lachman test exhibited no increased side-to-side difference and a firm endpoint.

**Conclusions:**

Extra-articular anterolateral stabilisation of the knee in patients having anterolateral knee instability combined with PVNS is a safe and efficient surgical treatment option yielding significant clinical improvement as well as subjective joint stability.

## Background

Diffuse pigmented villonodular synovitis (PVNS) is a rare proliferative disease (prevalence of 1.8 cases per 1 million) with recurrence rates as high as 48% after surgical procedures [1]). PVNS mainly affects patients between 20 and 40 years of age with a slight female predilection [1, 2]. However, there is no difference between the genders considering age distribution [2]. Current research suggests the knee being the most commonly affected site for PVNS in 50–75% of cases [2–4]. Common symptoms of diffuse PVNS are slowly progressive soft tissue swelling surrounding the joint, impaired range of motion and recurring joint locking. As PVNS frequently presents with a refractory pattern, its long term management can be difficult, since it often includes complicated and demanding courses of disease as well as a high socioeconomic burden.

Chronic progressive tissue degeneration (cartilage, ligaments etc.) can be a pattern of PVNS due to the chronic proinflammatory and catabolic microenvironment within the joint [4, 5]. Therefore, in addition to synovectomy, reconstructive surgery may also have to be carried out to preserve the structural integrity of the knee [6, 7]. However, due to impaired fibrous healing of intra-articular transplants, conventional ACL reconstructive techniques are mostly contraindicated [8]. Therefore, alternative approaches such as the Lemaire procedure have recently gained interest, since these novel techniques might help to overcome current limitations in ACL reconstruction in PVNS [7, 9], which include impaired graft healing and graft failure [8, 10]. Biomechanically, lateral extra-articular tenodeses can reduce rotatory laxity of the knee [11]. Therefore, this technique may be considered to restore native joint kinematics in a subset of patients after careful consideration of chances and risks.

As far as we are aware, we describe the first case of a young patient with diffuse PVNS and chronic anterolateral instability to undergo an isolated modified Lemaire extra-articular stabilisation of the knee.

## Case presentation

**Fig. 1:**
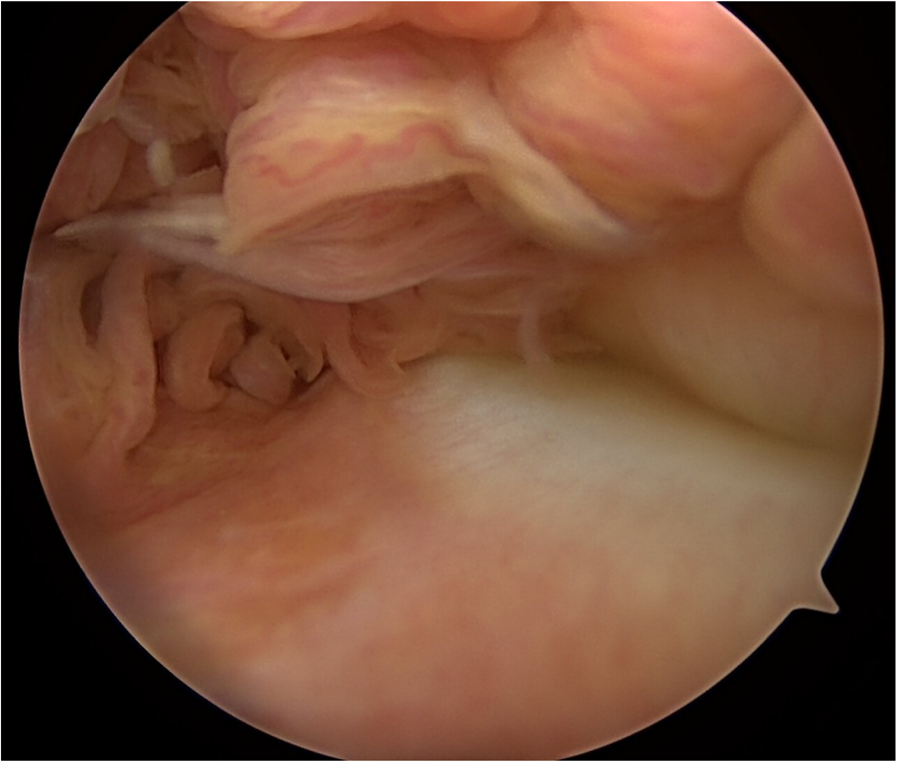
Diagnostic arthroscopy showing exhuberant intra-articular synovial proliferation, synovial inflammation and papillary projections, which are typical of diffuse PVNS

A 21-year-old patient, amateur basketball player, presented initially in 2014 with a diffuse swelling of the knee. The swelling occurred intermittently over approximately two months without an obvious trigger. Furthermore, the patient reported pain after extended basketball training sessions. Magnetic resonance imaging (MRI) of the knee revealed significant articular effusion and villous synovial proliferation**.** A histological biopsy was taken which revealed chronic synovialitis, villous hyperplasia and hemosiderin stained giant cells. Arthroscopic findings (Fig. 1) confirmed the diagnosis of diffuse PVNS of the knee and an intact ACL. Subsequently, an arthroscopic total synovectomy was performed in order to slow down the progression of cartilage degeneration as well as to restore range-of-motion, reduce pain, and joint instability.

**Fig. 2:**
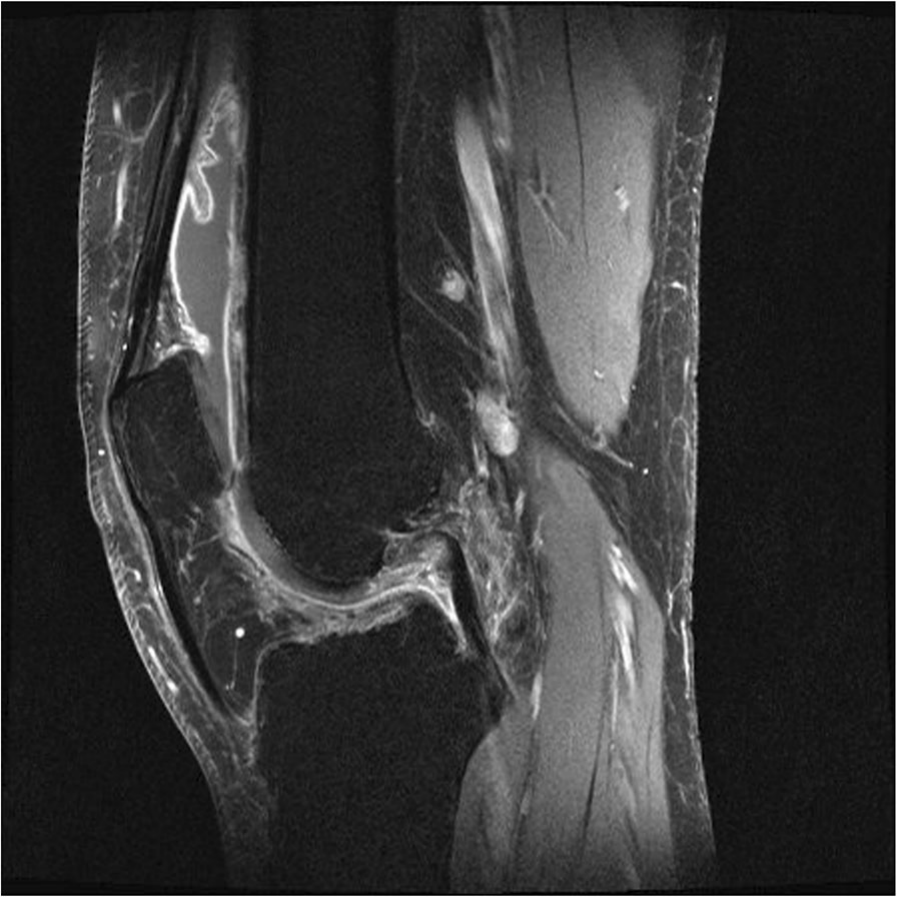
Saggital T2 MRI of the knee with fat saturation showing extensive intra-articular inflamed synovia and a ruptured ACL

**Fig. 3:**
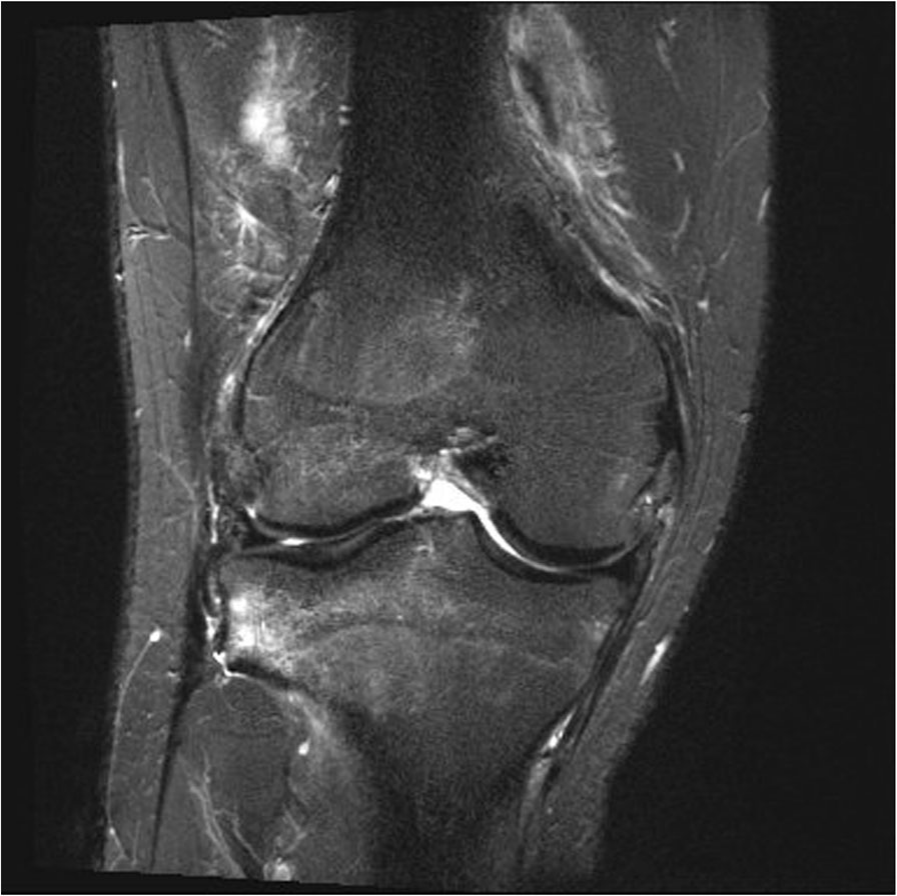
Coronal T2 MRI of the knee with fat saturation showing a ruptured ACL and high intensity signalling in the notch

After 6 asymptomatic months, the patient started suffering repeated knee distortions due to persistent anterolateral instability in his knee joint. A follow-up MRI confirmed the recurrent PVNS and an ipsilateral ACL rupture (Figs. 2 and 3). At this point, the patient opted for intensive physical therapy to stabilise the joint. During this time, he described an acute rotational trauma while exiting his car resulting in a small bucket handle tear in the white-white zone of the medial meniscus of the ipsilateral knee. Owing to coexisting degenerative changes of the dislocated fragment of the medial meniscus, anatomical reduction of the meniscus was not reasonable. Therefore, a re-synovectomy with a partial meniscectomy was conducted. 7 months later a similar mechanism of injury caused a re-rupture of the medial meniscus. Again a bucket handle tear was present, this time at the basis of the meniscus running from the pars intermedia to the posterior horn. Under the intention of restoring the structural integrity of this young patient’s knee another synovectomy and meniscus repair was undertaken. However, the patient suffered persistent giving away phenomena and was not able to further perform sports or properly work as an electrician.

The remaining therapeutic options to address the patient’s persisting anterolateral instability were re-assessed within a multidisciplinary consultation with oncologists, radiologists and orthopaedic surgeons. Due to the chronic intra-articular effusion and synovitis, an extra-articular stabilisation of the knee was suggested.

Pre-operative clinical examination of the right knee revealed minimal muscular atrophy and a considerable joint effusion compared to the healthy knee. The patient reported tenderness during palpation over the medial joint line. His active range of motion was unrestricted. During the Lachman test the knee exhibited greater anterior translation than the contralateral knee without a firm endpoint. The knee presented a grade 3 pivot shift. There was no posterior drawer effect. The valgus and varus stress tests were negative at 0° and 30° degrees flexion. Steinmann I was discretely positive for the medial meniscus.

**Fig. 4:**
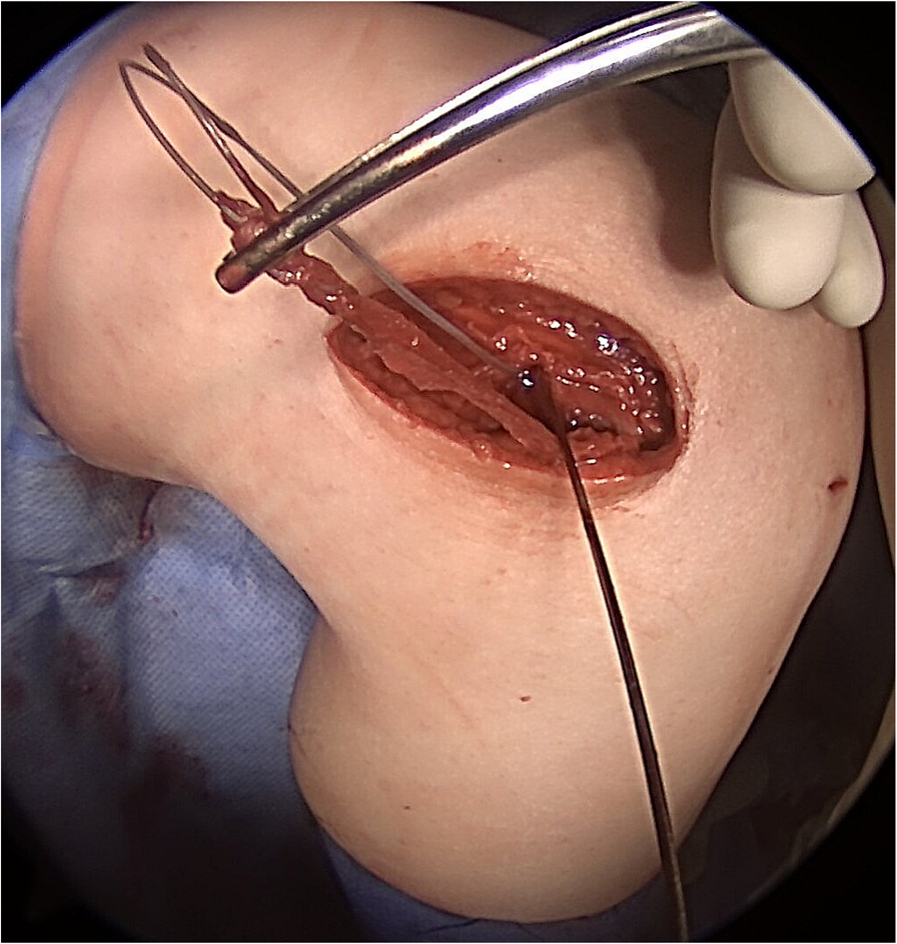
First intra-operative image. The tractus band transplant was inserted underneath the LCL and pulled into the socket

**Fig. 5:**
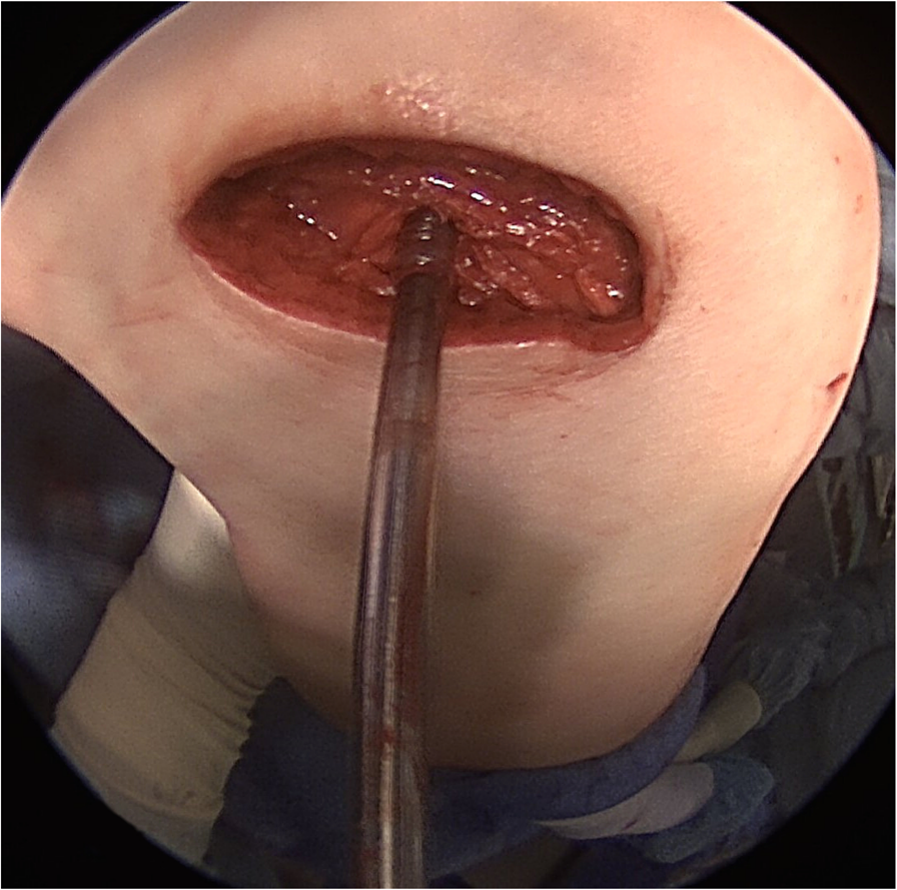
Second intra-operative image. The lower leg was secured in 70° flexion and neutral position to ensure the optimal tension of the transplant during the anchoring. Finally, the transplant was tensed gradually until the pivot shift was suspended and was subsequently fixated with an interference screw at 40 N

After conventional pre-operative preparations, surgery was performed. Briefly, a renewed medial meniscus suturing was performed arthroscopically, as again a complex tear of the posterior horn was present. All-inside suturing using a meniscus suturing device was carried out. Successively, the tractus iliotibialis was exposed and the lateral epicondyle was identified through palpation. The tractus was exised 0.5 cm dorsal of the lateral epicondyle, followed by its split parallel to the direction of the fibres, until yielding slightly proximal of Gerdy’s tubercle. A 10x1cm strip was separated and armed with a Fiberwire with a baseball-stitch technique. The lateral collateral ligament (LCL) was exposed underneath the tractus iliotibialis. A 6 mm socket was made proximal and slightly posterior to the origin of the LCL. The tractus band transplant was inserted underneath the LCL and pulled into the socket (Fig. 4). The lower leg was secured in 70° flexion and neutral position to ensure the optimal tension of the transplant during the anchoring. Finally, the transplant was tensed gradually from 20 N, 30 N to finally 40 N until the pivot shift was suspended and was subsequently fixated with an interference screw (Fig. 5).

Post-operatively, the clinical examination revealed a grade 0 pivot-shift test compared to the contralateral side. The Lachman test exhibited no increased side-to-side difference. The patient was compliant with the standard functional aftercare for meniscal suturing (range-of-motion limitation of 45° flexion for 3 weeks, followed by a limit of 90° flexion for further 3 weeks). In the latest follow-up examination at 12 months, the patient reported no further subjective instability and no swelling. He was able to perform moderate sports such as jogging and fully returned to work. In the clinical examination the patient showed dynamic joint stability during walking. The patient showed a grade 0 pivot-shift test compared to the contralateral knee. The Lachman test exhibited little increased side-to-side difference (Grade 1). Clinically, the patient showed no signs of progression of structural damage in the knee.

## Discussion

In this case, we conducted an extra-articular reconstruction resulting in a clinical and functional improvement in the condition of the patient’s knee. Usually, an intra-articular ACL reconstruction is contraindicated in patients with degenerative and pro-inflammatory intra-articular joint diseases such as PVNS. This condition was compounded by the patient describing chronic anterolateral instability with multiple distortions, causing additional structural damage to the knee. Therefore, this report suggests that isolated extra-articular anterolateral stabilisation of the knee in patients having anterolateral knee instability combined with PVNS presents a safe and efficient surgical treatment yielding in clinical improvement as well as subjective joint stability.

Extra-articular anterolateral reconstruction can be performed as an isolated surgery or to augment an intra-articular reconstruction [7, 12]. Combined extra- and intra-articular reconstruction may protect the intra-articular graft during the healing process and providing secondary restraint to the pivot-shift phenomenon [12–14]. Historically, most isolated extra-articular procedures performed in the 1970s–1980s were shown to provide restraint to the pivot-shift and Lachman manoeuvres. However, they were liable to stretch out in the long-term [15]. Additionally, these procedures were not able to address increased anterior tibial translation [9, 16]. With the recent resurgence of interest in extra-articular ACL reconstruction procedures, current literature suggests isolated extra-articular reconstruction may be performed in a small subset of patients with high-grade rotational laxity and an increased risk of failure with ACL reconstruction, such as revision cases [17–19].

The Lemaire procedure was first described in 1967 to treat ACL deficient knees with chronic instability. A long graft of fascia lata still attached to Gerdy’s tubercle was passed below the LCL [13, 20], and then attached through a femoral bone tunnel. In the following, the graft was reinserted under the LCL before being attached at another bony tunnel under Gerdy’s tubercle [9, 17]. This process has been simplified by securing the graft in the femur utilizing an interference screw as described by M. Wagner and A. Weiler [7]. Great care should be taken to perform pre-operative examination under anaesthesia before securing a tourniquet since the pressure of the tourniquet causes tension of the iliotibial tract which can stabilise the pivot-shift, therefore, causing a false-negative test [7].

Recent literature examining the biomechanics of extra-articular tenodesis suggests graft tension exceeding 20 N may increase lateral contact pressures and lead to overconstraint in internal rotation [21–23]. Specifically, one study by Inderhaug E. et al. comparing anterolateral procedures in combination with ACL reconstruction shows that grafts with 20 N of tension best restore native knee kinematics. In this case, an isolated anterolateral tenodesis was carried out in absence of an anterior cruciate ligament. During surgery tensioning was carried out using a spring scale. The pivot-shift was reduced at 20 N, however was still present. At 40 N the pivot-shift was suspended completely. In this set-up suspension of the pivot-shift was given higher priority compared to possible overconstraint of the knee in deep flexion degrees, as persistent instability might cause secondary damage to cartilage and menisci of the knee. In this case, our patient currently does not exhibit symptoms of overconstrained internal rotation or increased lateral tibiofemoral pressures.

The pivot-shift phenomenon is considered to have a multi-factorial pathogenic background in many cases. Contributing factors may include ligamentous laxity, altered bone morphology, and other soft tissue injuries [24–26]. Succeeding careful consideration of these factors, the pivot-shift test can be considered a reliable examination to evaluate rotational laxity. An increase in the grade of pivot-shift has been noted in injuries of the lateral structures in the ACL- deficient knee [24]. Additionally, some studies have shown the pivot-shift test can remain positive after intra-articular ACL reconstruction in a subset of patients, since even successful ACL reconstruction may fail to restore native knee functionality in regards to rotational abnormalities [17, 27, 28].

It is well established that timely ACL reconstruction reduces the risk of medial meniscal ruptures [29]. In the present case, PVNS presented a contraindication for ACL reconstruction due to the locally destructive and inflammatory nature of the disease. The pro-inflammatory joint microenvironment maintained by PVNS may have impaired the graft healing and increased the risk of graft failure [8, 10]. Additionally, the patient presented a high-grade anterolateral rotational instability. We presume that this chronic instability caused the recurrent medial meniscal tears over the course of the disease [29]. Therefore, despite the limitations which are historically described in isolated extra-articular reconstruction, in this case, a modified Lemaire extra-articular stabilisation of the knee was the most suitable procedure to restore knee kinematics and provide joint stability. In the future, it would be interesting to see studies showing the influence of isolated extra-articular stabilisation or combined intra- and extra-articular reconstruction on meniscal pathologies [13].

## Conclusion

Extra-articular anterolateral reconstruction of the knee is a feasible alternative for patients with anterolateral knee instability combined with PVNS yielding in clinical improvement and subjective joint stability. Diffuse PVNS of the knee can be a complex disease which can be associated with a long-drawn-out clinical course with multiple complications. In the present case, the objective of the treatment was to prevent further recurrence and restore the structural integrity of the patient’s knee. Especially in light of the patient’s young age, the long-term goal was to forestall the indication for knee arthroplasty.

### Summary

The present manuscript describes the case of a young patient with diffuse PVNS of the knee. We hereby show that an extra-articular stabilisation of the knee can be utilized for managing patients who imply contraindications for intra-articular reconstruction.

## Abbreviations

ACL: Anterior cruciate ligament
LCL: Lateral collateral ligament
PVNS: Pigmented villonodular synovitis
